# Percutaneous Peripheral Nerve Stimulation in Chemotherapy-Induced Neuropathy: A Case Report

**DOI:** 10.3390/reports8030133

**Published:** 2025-08-01

**Authors:** Sara Mogedano-Cruz, Carlos Romero-Morales, Mónica de la Cueva-Reguera, Kristin L. Campbell, Pablo Herrero

**Affiliations:** 1 Department of Physiotherapy, Faculty of Medicine, Health and Sports, European University of Madrid, Villaviciosa de Odón, 28670 Madrid, Spain; carlos.romero@universidadeuropea.es (C.R.-M.); monica.delacueva@universidadeuropea.es (M.d.l.C.-R.); 2 Department of Physical Therapy, Faculty of Medicine, University of British Columbia, Vancouver, BC V6T 1Z4, Canada; kristin.campbell@ubc.ca; 3 Institute for Health Research Aragon, Faculty of Health Sciences, University of Zaragoza, 50009 Zaragoza, Spain; pherrero@unizar.es

**Keywords:** chemotherapy-induced peripheral neuropathy, percutaneous electrical nerve stimulation, oxaliplatin, neuropathic pain, colon cancer

## Abstract

**Background and Clinical Significance**: Chemotherapy-induced peripheral neuropathy (CIPN) is a frequent and limiting complication of oncological treatment, particularly in patients receiving oxaliplatin. Its onset can significantly affect the quality of life and compromise the continuity of the antineoplastic therapy. Due to the limited efficacy of available pharmacological therapies, percutaneous electrical nerve stimulation (PENS) has been proposed as a non-invasive alternative for symptom management. **Case presentation**: We report the case of a 75-year-old woman with colorectal adenocarcinoma who developed CIPN following oxaliplatin administration. She underwent a 12-week course of PENS targeting the median nerve, with weekly sessions conducted without interruption of chemotherapy and without adverse effects. The patient showed progressive improvement in neurosensory symptoms, as measured by the EORTC QLQ-CIPN20 questionnaire. Quantitative sensory testing revealed normalization of thermal and vibratory sensitivity and improved mechanical detection thresholds. The cumulative oxaliplatin dose was maintained throughout treatment. **Conclusions**: PENS may offer an effective and safe therapeutic option for managing CIPN, enabling symptom control without compromising oncological treatment. This case supports the need for controlled clinical trials to confirm efficacy and establish standardized protocols.

## 1. Introduction and Clinical Significance

Chemotherapy is one of the most common treatments for cancer. Although its goal is to eliminate rapidly growing malignant cells, it also affects healthy cells [1]. This collateral damage often results in a clinical condition known as chemotherapy-induced peripheral neuropathy (CIPN), one of the most prevalent and debilitating complications of oncological treatment [2]. CIPN not only compromises patients’ quality of life but may also lead to dose reductions or even discontinuation of chemotherapy, directly impacting oncological prognosis. The long-term consequences of CIPN can be particularly disabling, often resulting in persistent functional impairment and significant psychological distress [3].

Among cytotoxic agents, oxaliplatin is particularly associated with the development of CIPN. This third-generation platinum compound, primarily used in the treatment of colorectal cancer and other gastrointestinal tumors, has significantly advanced the treatment of neoplasms and improved survival rates since its FDA approval in 2002. However, its use is limited by the high incidence of associated neurotoxicity [4].

Oxaliplatin-induced neurotoxicity includes both acute and chronic impairments. In the short term, up to 90% of patients experience neuropathic symptoms shortly after the first infusion [5,6], and the prevalence one month after finishing chemotherapy approaches 68% [6], persisting in approximately 41% of patients beyond three months [7].

These manifestations typically involve a progressive distal sensory neuropathy with a “stocking glove” distribution, characterized by dysesthesia, allodynia, hyperalgesia, and proprioceptive deficits. These symptoms reflect damage to Aβ, Aδ, and C fibers, which are critical for transmitting mechanical, thermal, and pain sensations. In severe cases, symptoms may persist for months or even years after chemotherapy, leading to significant functional disability [8,9].

Despite its clinical relevance, the therapeutic management of CIPN is limited to modifying the chemotherapy regimen, dose reduction, or drug suspension, which may compromise oncological efficacy [3]. Pharmacological treatments usually provide modest relief and are frequently associated with adverse effects [1,10,11]. Consequently, the search for effective, safe, and non-interfering therapeutic alternatives is a priority.

In this context, percutaneous peripheral nerve stimulation (PENS) has emerged as a novel, minimally invasive therapeutic alternative for managing neuropathic pain. This technique involves applying an electrical current through a percutaneously inserted electrode targeting a peripheral nerve [9]. Based on the gate control theory of pain modulation proposed by Melzack and Wall, PENS induces an inhibitory effect on pain transmission pathways at both spinal and supraspinal levels [12,13]. Evidence accumulated from various neuropathic pain syndromes suggests that this approach can improve physical function, reduce pain intensity, and enhance quality of life [14,15,16,17,18].

Specific PENS of the median nerve, which innervates the palmar aspect of the fingers, a region frequently affected in CIPN [7], offers significant therapeutic potential. Although its use in this neuropathy is still emerging, the underlying pathophysiological rationale and preliminary clinical evidence support its promising role [19].

This report presents the first documented clinical case of successful CIPN management via PENS. Its clinical relevance is reinforced by the growing number of cancer survivors requiring effective and lasting interventions for persistent functional sequelae that compromise quality of life [4].

## 2. Case Presentation

### 2.1. Patient Information

A 75-year-old right-handed Caucasian woman sought oncology consultation to begin treatment after being diagnosed with colorectal adenocarcinoma. As part of the therapeutic protocol, a chemotherapy regimen with oxaliplatin was prescribed at a dose of 145 mg intravenously, administered every 15 days.

Before initiating chemotherapy, the patient’s neurological examination was unremarkable, with no clinical signs or history suggestive of peripheral sensory or motor involvement. Baseline nerve conduction studies and electromyography showed no abnormalities. Laboratory tests revealed normal renal, hepatic, and metabolic function, except for a mild normocytic normochromic anemia (hemoglobin 11.6 g/dL, hematocrit 35.9%).

Her past medical history included hypertension and dyslipidemia, both controlled with beta-blocker therapy. Apart from dexamethasone as part of the oncological regimen, no other chronic medications were recorded.

### 2.2. Clinical Findings

After the first oxaliplatin infusion, the patient developed dysesthesias in both hands triggered by cold exposure, accompanied by grade 1 paresthesia. These symptoms were transient and resolved spontaneously within four days. During the perichemotherapy period, symptoms were mitigated by avoiding cold exposure. No motor deficits or signs of central or autonomic involvement were noted.

### 2.3. Patient Evolution

The patient received oxaliplatin chemotherapy cycles every 15 days. Treatment continued regularly, except for the fourth cycle, which was delayed one week due to mild neutropenia. After the third cycle, a gradual decrease in neuropathic symptoms in the hands was observed. However, after the fifth cycle, the patient developed new symptoms, including paresthesias in both feet and oropharyngeal dysesthesia upon ingestion of cold liquids. Subsequent neurological examinations remained normal, with no changes in muscle strength, deep tendon reflexes, or coordination.

During the intermediate cycles, laboratory findings remained largely within normal limits, although a progressive decrease in creatinine levels was noted, reaching a value of 0.41 mg/dL in the third cycle. From the seventh cycle onward, hematological studies showed a reduction in the count of leukocytes, neutrophils, erythrocytes, and platelets, which was managed with close monitoring without requiring treatment suspension.

### 2.4. Diagnostic Assessment

The patient’s clinical presentation was consistent with oxaliplatin-induced peripheral neuropathy, initially presenting as an acute, transient, cold-related form in the upper limbs and later progressing to the lower limbs. The chronology, symptom distribution, and temporal association with chemotherapy administration allowed for the clinical diagnosis of CIPN.

The initial prognosis was considered favorable based on the patient’s overall clinical stability. However, the emergence of neuropathic symptoms highlighted the need to explore alternative therapeutic interventions.

### 2.5. Therapeutic Intervention

At the beginning of the intervention, the patient reported symptoms exclusively in the upper limbs, primarily affecting hand function. For this reason, a physiotherapeutic intervention based on PENS of the median nerve was applied, aiming to modulate CIPN symptoms in the hands. The median nerve was specifically selected for stimulation due to the sensory distribution of the patient’s symptoms, which were predominantly localized to the palmar aspects of the fingers innervated by this nerve.

**Figure 1:**
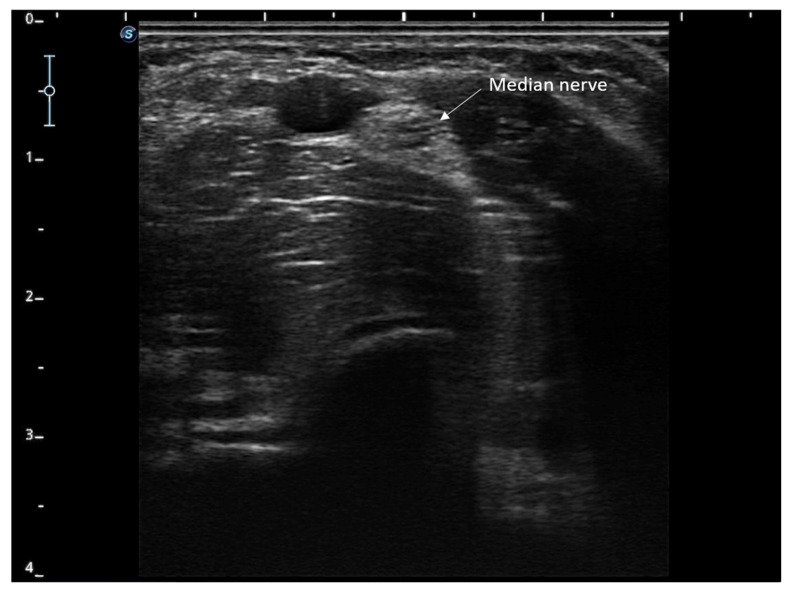
Localization of the median nerve in the anterior region of the elbow.

The intervention was performed under ultrasound guidance to precisely locate the median nerve in the anterior region of the elbow (Figure 1). Once identified, a sterile needle was inserted until the epineurium was reached and connected to a peripheral nerve stimulator (Physio Invasiva^®^, CEO120; PRIM Physio, Madrid, Spain). A biphasic rectangular current was applied at a frequency of 1 Hz, a pulse width of 200 µs, and an intensity adjusted to the maximum threshold tolerated by the patient (1.3 mA). Stimulation was maintained for 30 min per session [12,20,21,22].

During stimulation, the patient reported a mild cramping sensation upon nerve contact and involuntary contractions of the flexor forearm muscles, indicating an adequate response. Treatment was administered once a week over 12 weeks, without complications and was well tolerated.

### 2.6. Study Variables and Follow-Up

Quality of life was assessed using the European Organization for Research and Treatment of Cancer (EORTC) questionnaire for evaluating CIPN (QLQ-CIPN20). This questionnaire consists of 20 items, divided into three subscales: sensory symptoms (9 items), motor symptoms (8 items), and autonomic symptoms (3 items). Each item is scored on a Likert scale from 1 to 4, where 1 represents the absence of symptoms and 4 indicates maximum severity [23,24].

Neural sensitivity was measured using the quantitative sensory testing (QST), which included three types of tests to assess different aspects of the somatosensory function [25]. First, mechanical detection thresholds (MDTs) were evaluated using the “up/down” method with von Frey monofilaments [26], which apply forces ranging from 0.02 to 10 g. Each filament was applied for approximately one second on the palmar surface of the distal interphalangeal joint of the index finger, starting with the one with the lowest force. The procedure was then repeated with increasingly stronger filaments (0.02, 0.07, 0.5, 2, and 10 g) until the patient reported perceiving the tactile stimulus [27]. The force corresponding to the first detected filament was applied three times to confirm the perception, and the average value of these three measurements was recorded as the tactile threshold. Subsequently, vibration thresholds (VTs) were assessed using the NerveCheck^®^ device (Phi Med Europe, Barcelona, Spain). Nine random stimuli of different intensities (none, light (2.7 V), moderate (4.2 V), and strong (6.4 V)) were applied to the palmar surface of the distal interphalangeal joint of the index finger, and the patient was asked to report whether vibration was perceived [27]. Finally, temperature detection thresholds (TDTs) were measured, also using the NerveCheck^®^ device. The thermode was placed on the palmar surface of the distal interphalangeal joint of the index finger, and a baseline temperature of 32 °C was established. For the cold detection test (CDT), five random stimuli of varying intensities were applied: none, light (22.4 °C), moderate (17.8 °C), and strong (9.8 °C). For the heat detection test (WDT), five random stimuli were applied with the following varying intensities: none, light (37 °C), moderate (39.4 °C), and strong (44.7 °C). The patient was instructed to indicate when a temperature change was perceived [27].

Finally, the cumulative dose of oxaliplatin (in mg) was recorded, obtained from the oncologist’s treatment records.

These variables were assessed at three different time points: prior to the PENS treatment period, after 4 weeks, and after 12 weeks of treatment.

### 2.7. Results

The first evaluation was conducted one week after the administration of the second oxaliplatin cycle. At baseline, quality of life assessed with the EORTC QLQ-CIPN20 questionnaire revealed significant neuropathic impairment, with high scores in tingling (3/4), stabbing pain (3/4), and cramps (2/4) in the hands, as well as difficulties handling small objects (2/4).

After four weeks of intervention, an improvement in quality of life was observed, with stable scores for tingling (3/4) and cramps (2/4), and resolution of stabbing pain and functional difficulties. At twelve weeks, symptoms continued to improve, with a further reduction in tingling (2/4) and sustained absence of stabbing pain and functional difficulties.

Regarding neural sensitivity measured by quantitative sensory testing, baseline assessment revealed decreased vibration thresholds (10/12) and significant dysfunction of Aδ and C fibers, reflected in a low warm detection threshold (2/6). Cold detection thresholds were within normal limits (6/6), and mechanical detection thresholds indicated decreased protective sensation. After four weeks of treatment, vibration and warm detection thresholds had normalized to maximum scores (12/12 and 6/6, respectively), with no changes in cold detection thresholds or mechanical detection thresholds. By week twelve, improvements were maintained in vibration, warm, and cold detection thresholds, and mechanical detection thresholds progressed from impaired protective sensation (baseline) to reduced light-touch sensitivity (12 weeks), reflecting a gradual recovery of sensory function.

The cumulative dose of oxaliplatin remained constant at 145 mg throughout the intervention period, indicating that a reduction in chemotherapy dosage due to neuropathy was not necessary. The patient demonstrated optimal adherence to the treatment, completing all scheduled sessions over the twelve-week period, with no adverse effects reported.

**Figure 2:**
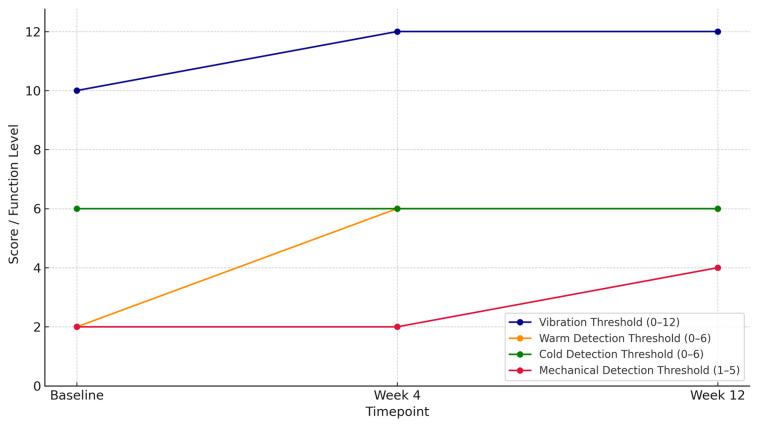
A graphical representation of the scores obtained in the Quantitative Sensory Testing (QST) at baseline, week 4, and week 12. Higher scores in the vibration threshold (0–12), warm detection threshold (0–6), cold detection threshold (0–6), and mechanical detection threshold (1–5) indicate improved sensory function.

To further illustrate the patient’s clinical trajectory, an additional descriptive intra-patient analysis was performed across the three assessment points. The most pronounced improvements were observed in stabbing pain and functional difficulties, which resolved completely by week 4 and remained absent at week 12, representing a 100% reduction from baseline. Tingling decreased from a severity score of 3 to 2 (a 33% reduction), whereas cramps remained stable. Regarding objective measures, vibration thresholds improved from 10/12 to 12/12 (complete normalization), and warm detection thresholds increased from 2/6 to 6/6. Mechanical detection thresholds evolved from decreased protective sensation to partial recovery, characterized by reduced light-touch sensitivity. Cold detection thresholds remained stable within the normal range at all time points. These temporal trends are summarized in Table 1 and Figure 2.

## 3. Discussion

CIPN is among the most frequent and debilitating complications of oncological treatments, particularly with oxaliplatin, known for causing persistent neurotoxicity that severely affects quality of life [28,29]. As survival rates among cancer patients improve, the incidence of CIPN is expected to increase, representing a growing challenge for clinicians and an additional threat to patients, as it can lead to reductions in chemotherapy doses, treatment interruptions, and even discontinuation of cycles [3]. Currently, CIPN is a complex disorder to manage, with few effective treatments available. In this context, PENS was selected as a non-pharmacological intervention due to its ability to modulate nerve activity and restore neurophysiological balance [12,13].

One of PENS’s main advantages is that it does not require the implantation of permanent devices, as needles are removed after each session, minimizing risks associated with invasive procedures [30]. In the presented case, the patient showed a significant improvement in quality of life, with a decrease in symptoms such as tingling, cramps, and stabbing pain in the hands, which had previously been highly reported. In addition, the results of the QST showed progressive normalization, with a recovery in the function of the Aβ, Aδ, and C fibers. These improvements in sensory parameters suggest that PENS may have a positive impact on nerve function altered by CIPN.

Another relevant finding in this case is that the patient was able to continue oxaliplatin treatment without the need for dose reduction, which is a crucial factor in managing the disease [10]. The ability to control symptoms without affecting the oncological treatment allows patients to maintain the efficacy of chemotherapy without compromising their quality of life.

Importantly, no adverse effects were reported throughout the intervention, and the patient’s adherence was optimal. These findings collectively suggest that PENS may contribute not only to symptomatic relief but also to an improved quality of life, without interfering with the ongoing chemotherapy regimen.

From a physiological perspective, the underlying mechanisms of PENS have been extensively studied in animal models [31], demonstrating reductions in central excitability of nociceptive neurons and a decrease in the release of excitatory neurotransmitters, such as glutamate, in the dorsal horn of the spinal cord. This effect appears to be related to the activation of central inhibitory pathways, which help restore the neurophysiological balance disrupted in CIPN [5]. The physiological mechanisms underlying the effects of PENS are multifaceted. At the peripheral level, stimulation may promote axonal regeneration and restore ion channel function in damaged nerve fibers. Centrally, PENS has been shown to reduce hyperexcitability of spinal dorsal horn neurons and to enhance inhibitory descending pathways [12,32,33,34]. These processes can modulate nociceptive transmission and contribute to pain relief. Moreover, this technique has already shown effectiveness in patients with neuropathies that do not respond well to conventional treatments and present hyperexcitability and symptoms such as allodynia or hyperalgesia [14,15,16,17,18].

Given the nature of a single-patient case report, the observations presented here are intended to be descriptive rather than to establish causal inferences. The outcomes should be interpreted with caution and viewed as preliminary clinical insights that may inform future hypothesis-driven research.

Despite these promising results, it is crucial to recognize that PENS remains an emerging technique, requiring further knowledge and standardization. Variability in stimulation parameters (intensity, frequency, duration) generates uncertainty regarding optimal application [35,36,37].

Additionally, given the chronic nature of CIPN, the lack of long-term follow-up is another limitation in thoroughly assessing the sustainability of the observed effects. Although symptom improvement was observed following a 12-week PENS protocol, prolonged follow-up is necessary to determine whether these benefits are maintained over the long term.

This case represents the first report documenting the success of PENS in treating medically refractory CIPN. However, controlled and randomized studies are needed to confirm these findings in a more diverse and representative population.

## 4. Conclusions

PENS appears to be a promising therapeutic option for treating CIPN, with preliminary results suggesting efficacy in improving neurosensory symptoms without interfering with cancer therapies. However, further clinical research is needed to validate these results and establish clear guidelines for their clinical application.

## Figures and Tables

**Table 1:** Descriptive evolution of the subjective and objective outcome measures. Higher scores in “Tingling”, “Cramps”, “Stabbing pain”, and “Functional difficulties” indicate greater symptom severity; thus, a decrease reflects clinical improvement. For “Vibration Thresholds”, “Warm Detection Thresholds”, and “Cold Detection Thresholds”, higher scores indicate better sensory function. In the case of “Mechanical Detection Thresholds”, the categories reflect different degrees of mechanical sensitivity based on the force required to elicit tactile perception. The observed progression from “decreased protective sensation” to “reduced light-touch sensitivity” suggests a recovery of mechanical sensory function.

Variable	Baseline	Week 4	Week 12	Change (Baseline–Week 12)	Percentage Change
Tingling (0–4)	3/4	3/4	2/4	−1	−33%
Cramps (0–4)	2/4	2/4	2/4	0	0%
Stabbing pain (0–4)	3/4	0/4	0/4	−3	−100%
Functional difficulties (0–4)	2/4	0/4	0/4	−2	−100%
Vibration Thresholds (0–12)	10/12	12/12	12/12	+2	+20%
Warm Detection Thresholds (0–6)	2/6	6/6	6/6	+4	+67%
Cold Detection Thresholds (0–6)	6/6	6/6	6/6	0	0%
Mechanical Detection Thresholds	Decreased protective sensation	No change	Reduced light-touch sensitivity	Partial recovery	—

## Data Availability

The original contributions presented in this study are included in the article. Further inquiries can be directed to the corresponding author.

## Abbreviations

The following abbreviations are used in this manuscript:

CIPN	Chemotherapy-induced peripheral neuropathy
FDA	Food and Drug Administration
PENS	Percutaneous peripheral nerve stimulation
EORTC QLQ-CIPN20	European Organization for Research and Treatment of Cancer questionnaire for evaluating CIPN
QST	Quantitative sensory testing
MDT	Mechanical detection thresholds
VT	Vibration thresholds
TDT	Temperature detection thresholds
CDT	Cold detection test
WDT	Heat detection test
